# Cake: a bioinformatics pipeline for the integrated analysis of somatic variants in cancer genomes

**DOI:** 10.1093/bioinformatics/btt371

**Published:** 2013-07-16

**Authors:** Mamunur Rashid, Carla Daniela Robles-Espinoza, Alistair G. Rust, David J. Adams

**Affiliations:** Experimental Cancer Genetics, Wellcome Trust Sanger Institute, Hinxton, Cambridgeshire, CB10 1HH, UK

## Abstract

**Summary:** We have developed Cake, a bioinformatics software pipeline that integrates four publicly available somatic variant-calling algorithms to identify single nucleotide variants with higher sensitivity and accuracy than any one algorithm alone. Cake can be run on a high-performance computer cluster or used as a stand-alone application.

**Availabilty:** Cake is open-source and is available from http://cakesomatic.sourceforge.net/

**Contact:** da1@sanger.ac.uk

**Supplementary Information:** Supplementary data are available at *Bioinformatics* online.

## 1 INTRODUCTION

The development of next-generation sequencing technologies has made it possible to generate more comprehensive catalogues of somatic alterations in cancer genomes than ever before. Software tools to find these variants deploy different mathematical approaches to interrogate the genome sequences of tumour/germline paired samples. For example, the variant detectors Bambino (Edmonson *et al.*, 2011) and VarScan 2 (Koboldt *et al.*, 2012) both identify somatic variants by comparing alternative allele frequencies between tumour and normal sequences. VarScan 2 uses a Fisher’s exact test and Bambino a Bayesian scoring model to identify somatic variants in paired samples. Other algorithms include CaVEMan (Stephens *et al.*, 2012) and SAMtools mpileup (Li *et al.*, 2009), which compute the genotype likelihood of nucleotide positions in tumour and normal genome sequences by use of an expectation-maximization method.

Putative raw variant calls made by these algorithms typically undergo further filtering. For example, known single nucleotide polymorphisms (SNPs) present in dbSNP (Sherry *et al.*, 2001) or in the 1000 Genomes project (The 1000 Genomes Project Consortium *et al.*, 2012), or sites with low mapping qualities are usually filtered from the final somatic call set. Validation rates ultimately depend on the stringency of this filtering of putative sites.

Intriguingly, applying different variant-calling algorithms to the same data often results in a set of only partially overlapping somatic single nucleotide variant (SNV) calls. To illustrate this phenomenon, we deployed four publicly available somatic variant-calling algorithms (Bambino, CaVEMan, SAMtools mpileup and VarScan 2) on a dataset composed of 24 human hepatocellular carcinoma tumour/germline exome pairs (Guichard *et al.*, 2012). Because this study reported 994 validated somatic variants identified using the independent CASAVA pipeline, we used these data to gauge the performance of each algorithm. This analysis revealed at best a 43.8% overlap between SNV calls made by any two of these widely used callers, and at worst a 6.45% overlap (Supplementary Table S1). Notable, however, was the fact that the majority of validated calls were identified by two or more algorithms, suggesting that a merging approach may improve both the sensitivity and accuracy of somatic variant calling. See the Supplementary Material for details.

In an effort to take advantage of existing software tools and to improve variant detection, we developed Cake (Supplementary Fig. S1). Cake is a fully configurable bioinformatics pipeline that integrates four single nucleotide somatic variant-calling algorithms (Bambino, CaVEMan, SAMtools mpileup, and VarScan 2) and deploys an extensive collection of fully customizable post-processing filtering steps. We show that the performance of Cake exceeds any one algorithm for somatic SNV detection, making it an optimal tool for cancer genome analysis.

## 2 IMPLEMENTATION

Cake is implemented in Perl, enabling the configuration, execution and monitoring of the four callers in a high-performance computing environment using a job scheduler. Alternatively, Cake can be configured to run in stand-alone mode on a single computer (See the User Manual on SourceForge for more details). The standard Cake workflow is to run all of the algorithms individually, merge the predicted SNVs reported by at least any two (Supplementary Fig. S2) somatic callers and then apply the post-processing filters. This configuration can, however, be easily adjusted as required (Supplementary Table S2). The existing choice of algorithms can also be modified using a template we provide. A package containing wrappers around the callers, the post-processing modules and an installation script is available for download.

## 3 RESULTS

To evaluate the performance of Cake, we used the aforementioned human hepatocellular carcinoma dataset composed of 24 exome tumour/germline pairs and two human breast cancer exomes for which we had genomic DNA for follow-up validation (Stephens *et al.*, 2012). The performance of each variant-calling algorithm was evaluated by running each one individually using their default settings and filtering the results using the post-processing filters implemented in Cake. The results are summarized in Table 1.

**Table 1.** Summary of the results of different somatic variant-calling algorithms and Cake on two human exome datasets

		Hepatocellular carcinoma (24 samples/842 validated sites)			Breast cancer (2 samples/264 validated sites)			
Calling strategy	Algorithms	Validated mutations identified (total)	Sensitivity (%)	Average number of variant calls per sample	Validated mutations identified (total)	Sensitivity (%)	Average number of variant calls per sample	Validation success rate (Sequenom) (%)
Single algorithms (after filtering)	Bambino	742	88.1	2503 ± 1070	248	93.9	3456 ± 324	
	CaVEMan	801	95.1	1072 ± 1055	(263)	(99.6)	(961 ± 90)	
	Mpileup	727	86.3	429 ± 226	181	68.6	329 ± 32	
	VarScan 2	805	95.6	926 ± 527	205	77.7	929 ± 91	

Cake	≥ any 2 callers	812	96.4	634 ± 299	254	96.2	613 ± 42	51.5
	≥ any 3 callers	794	94.3	270 ± 132	214	81.1	326 ± 50	81.7
	4 callers	652	77.4	168 ± 98	166	62.8	178 ± 42	88.3

### 3.1 Human hepatocellular carcinoma dataset

In their study, Guichard *et al.* (2012) experimentally validated 850 SNV positions, of which 8 were not covered by sequence reads following realignment leaving a target reference set of 842. Using Cake with an intersection of any two or more algorithms, 812 validated variants were retained (Supplementary Fig. S3), representing an overall sensitivity of 96.4%. An average of 634 variants was predicted per exome (Table 1). Cake outperformed the best single algorithm in terms of specificity and the number of variants reported per sample.

### 3.2 Human breast cancer exome dataset

Because the above analysis will favour callers that perform like CASAVA, and because we did not have DNA from the hepatocellular carcinomas for follow-up analysis to ascertain the false positive and negative rates, we next used exome data from two breast tumours for which whole genome amplified tumour and germline DNA was in hand. Using Cake and an intersection of any two or more callers, we made 1225 calls (per sample 613 ± 42), of which 254 were from a reference call set representing a subset of positions (264) covered by the capture baits where a somatic mutation had resulted in a non-synonymous change; a sensitivity of 96.2% (Table 1, Supplementary Fig. S4). Excluding CaVEMan, which was used in the original study, Cake again outperformed all other algorithms (Table 1).

To assess the specificity of the somatic variant calling by Cake, we used the Sequenom MassARRAY SNP genotyping platform on tumour and germline DNA samples. A total of 400 variants were randomly selected from the 1225 calls made by any two or more callers in the Cake pipeline, 200 from each sample. Two hundred and seventy variants were validated, including 95 somatic mutations confirmed in the original study, 111 somatic mutation that were not described previously and 64 germline variants (Supplementary Fig. S5). Importantly, we called variants in a greater target region than the original study by analyzing positions in 5′ and 3′ untranslated regions, and introns. Six additional non-synonymous SNVs were discovered and confirmed (Supplementary Table 3), including variants in *AKAP1*, *PCNT* and *RERE*, all of which have been implicated in cancer.

A further 400 variants were included as a true negative set resulting in a worst-case accuracy for Cake of 75.8% [Accuracy = (95 + 111 + 400)/(400 + 400)]. Although we used our default of at least any two callers as part of the aforementioned analysis, 88.3% of positions that validated as somatic variants were reported by all four algorithms used by Cake (Supplementary Fig. S5, Table 1). This indicates that merging predictions increases the probability of identifying true mutations. Thus, we demonstrate that Cake may be used to help prioritize somatic SNVs calls for follow-up validation.

## 4 SUMMARY

Here we describe Cake, a software tool integrating four somatic variant detection algorithms to call variants with higher accuracy and specificity than any one algorithm alone. Cake performs well on whole genomes, exomes and targeted next-generation sequencing data, as well as on both human and mouse samples. Cake is freely available to the research community.

## Supplementary Material

Supplementary Data
