# Supplementary material for: Cake: a bioinformatics pipeline for the integrated analysis of somatic variants in cancer genomes
Source: Bioinformatics. 2013 Jul 16;29(17):2208–10. doi: 10.1093/bioinformatics/btt371 (PMC3740632; doi:10.1093/bioinformatics/btt371)
Supplement: Supplementary Data [file supp_29_17_2208__index.html]

Cake: a bioinformatics pipeline for the integrated analysis of somatic variants in cancer genomes — Cake: a bioinformatics pipeline for the integrated analysis of somatic variants in cancer genomes — Cake: a bioinformatics pipeline for the integrated analysis of somatic variants in cancer genomes — Supplementary Data 

# Cake: a bioinformatics pipeline for the integrated analysis of somatic variants in cancer genomes

## 

files

**Files in this Data Supplement:**

- Supplementary Data - pdf file
- Supplementary Data - pdf file
