# Supplementary material for: Cake: a bioinformatics pipeline for the integrated analysis of somatic variants in cancer genomes
Source: Bioinformatics. 2013 Jul 16;29(17):2208–10. doi: 10.1093/bioinformatics/btt371 (PMC3740632; doi:10.1093/bioinformatics/btt371)

Cake: User Manual

rm8@sanger.ac.uk, cdre@sanger.ac.uk, ar12@sanger.ac.uk

June 4, 2013

Contents

1 Overview **2**

2 Cake workflow **3**

3 Download required components and dependencies **4**
- 3.1 System requirements . . . 4
- 3.2 Required tools installation . . . 4
  - 3.2.1 Automated installation . . . 5
  - 3.2.2 Manual installation . . . 7
  - 3.2.3 `samtools` . . . 7
  - 3.2.4 `tabix` . . . 7
  - 3.2.5 `vcftools` . . . 8
  - 3.2.6 Varscan 2 . . . 8
  - 3.2.7 Bambino . . . 8
  - 3.2.8 `cmake` (Prerequisite for SomaticSniper, version $\geq$ 2.8) . . . 8
  - 3.2.9 `samtools_0.1.6` (Prerequisite for SomaticSniper) . . . 8
  - 3.2.10 SomaticSniper . . . 8
- 3.3 Perl modules . . . 9
  - 3.3.1 DBI . . . 9
  - 3.3.2 DBD::MySQL . . . 9
  - 3.3.3 YAML . . . 10
  - 3.3.4 File::Grep . . . 10
  - 3.3.5 Statistics::Descriptive . . . 10
  - 3.3.6 Text::NSP . . . 10
- 3.4 BioPerl and Ensembl APIs . . . 10
  - 3.4.1 BioPerl . . . 11
  - 3.4.2 Ensembl Core API . . . 11
  - 3.4.3 Ensembl Variation API . . . 11
  - 3.4.4 Ensembl Tools . . . 11
- 3.5 Update your `PERL5LIB` environment variable . . . 11
- 3.6 Additional files . . . 12
  - 3.6.1 Necessary files . . . 12
  - 3.6.2 Optional files . . . 12
  - 3.6.3 Ingredients check . . . 13

4 Running Cake **13**
- 4.1 Sample information file . . . 14
- 4.2 Run modes . . . 14
- 4.3 Run Cake . . . 14
- 4.4 General parameters . . . 15
- 4.5 Variant calling parameters . . . 15
- 4.6 Variant filtering flags and parameters . . . 16
- 4.7 Output files . . . 18

5 Example run **19**

1 Overview

This document describes the installation and running of the Cake software pipeline, which detects somatic variants from next-generation sequencing data. Descriptions of the required software tools and modules are given in detail, together with extensive details on the installation process. This document also has hyperlinks to useful sites within the text. The Cake workflow diagram in **Section 2** describes the different variant-calling and filtering components of Cake.

Since the pipeline uses multiple external software tools, the installation "recipe" does look somewhat daunting. If Cake is installed in an environment rich with bioinformatics tools, then a number of the required components may already be present. We have tried to simplify the installation procedure by providing a simple and customisable installation script. This installation script allows users to install all required software tools and Perl modules and update necessary software paths in the Cake configuration file (Automated installation, **Section 3.2.1**). Additionally, we have sought to try and provide as clear and concise instructions as possible where tools need to be installed from scratch. Once all required software has been successfully installed and Perl environments are updated, users can jump to the example run section (Example run, **Section 5**). We also provide a set of test data that can be used to check that Cake has been properly configured and runs correctly.

Cake can be run in different modes and various architecture types. In addition to traditional variant calling, Cake comes with a number of variant filtering modules to increase specificity and help prioritise variants for functional validation. **Section 4** contains a detailed description of the different running modes and its customizable parameters.

In this User Manual we have tried to describe the Cake installation, configuration, different run modes and filtering parameters. Please read these sections for a better understanding of the different modules. However, if you cannot wait to taste Cake, please jump to example run section (**Section 5**), where we have carefully designed a small test case to run Cake. It uses an automated dependency resolve script (See **Section 3.2** for details) to download, install and configure all the necessary software/modules as well as other related test files to run Cake, and configures it for the provided test files. Please remember to modify the configuration file to suit your system and data. Also update the environment variables as mentioned in the automated installation process.

2 Cake workflow

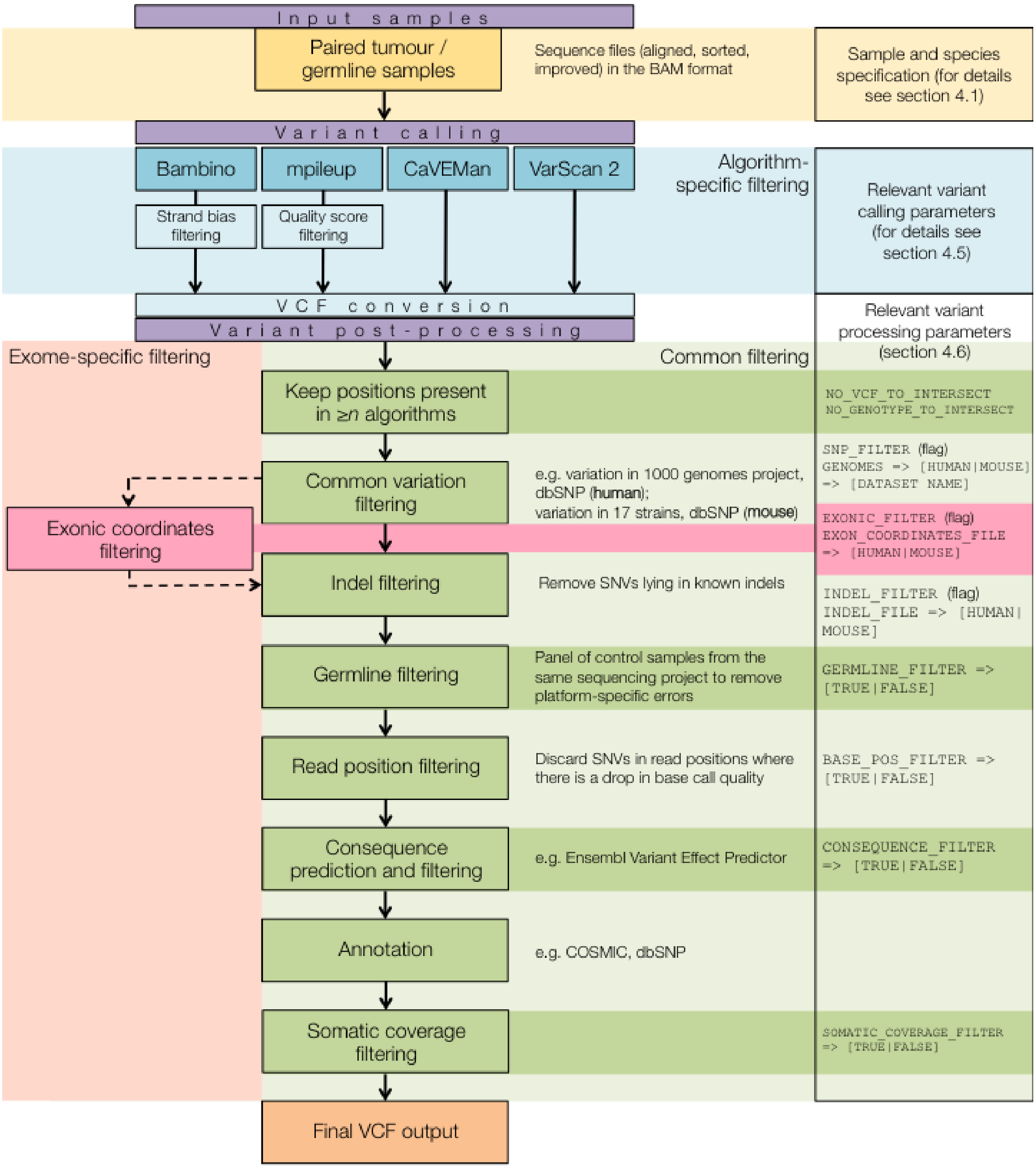

Cake is freely available for academic use and can be downloaded from the SourceForge download page: http://sourceforge.net/projects/cakesomatic/files/latest/download
Simply download the source code and decompress it:

```
mkdir DIR_TO_DOWNLOAD_CAKE (If it doesn't already exist)
cd DIR_TO_DOWNLOAD_CAKE
wget http://sourceforge.net/projects/cakesomatic/files/Cake_1.0.tar.gz
tar xfz Cake_1.0.tar.gz
```

Running the Cake pipeline is quite straightforward; however, there are some packages and tools that need to be downloaded prior to running an analysis. The software tools and Perl modules that Cake uses are freely available and can be easily configured. In the following sections we provide a list of instructions to install these tools. We have done our best to make these instructions as detailed as possible, and have been tested on the following OSX and Linux distributions:

**Operating System**	**Version**
Linux (Ubuntu)	10.04.4
Linux (Ubuntu)	10.04
Linux (Debian)	5.0.10
Max OSX	Snow Leopard 10.8.0
Max OSX	Lion 10.7.5
Max OSX	Mountain Lion 10.8
Distributed Architecture	LSF

3 Download required components and dependencies

For variant calling and filtering, Cake requires a number of standard bioinformatics tools (e.g. `vcftools`, `samtools`). All of these are open source/freeware and can be easily downloaded, installed and configured. Once installed, Cake looks up the software/executable file paths from a configuration file, located in

```
/{PATH_TO_CAKE}/trunk/perl/modules/SomaticCallerConfig.pm
```

From now on, we will refer to this file as `SomaticCallerConfig.pm`. **Section 3.2** will discuss how to install these tools and set their updated corresponding path in the configuration file.

3.1 System requirements

Mac OSX Users need to have Xcode installed, which is freely available from the Apple App Store. After downloading it, start Xcode, go to Xcode -> Preferences -> Downloads and install the component *Command Line Tools*. All relevant tools will be placed in `/usr/bin` and you will be able to use it without further requirements.

Linux Users need GNU `make` to compile and install most of these modules, which can be dowloaded from GNU Linux download page . After you have installed `make`, please add the path to your `$PATH` variable (in `~/.bashrc`, `~/.cshrc` or `~/.bash_profile`).

3.2 Required tools installation

Required software tools can be downloaded, installed and configured in two different ways:

1. Automated installation
2. Manual installation

Before you follow any of these approaches, please check if you have any of the following tools already in your `PATH` by typing 'which [tool-name]', e.g. `which samtools`. If a locally-installed copy is available for a tool, please update the bin/executable path for that tool in the `SomaticCallerConfig.pm` configuration file. If the automated installation script installs everything with no warnings (check the installation output in `{SOFTWARE_INSTALLATION_PATH}/Software_Installation_Status.txt` or the terminal output of the automated installation script), then you are ready to run the Cake pipeline and do not need to refer to the manual installation section. If one or more ingredients fail in automated installation, then please refer to the manual installation for those software/modules only.

3.2.1 Automated installation

This is a simple Perl script to install the necessary software and Perl modules. It updates the paths in `SomaticCallerConfig.pm`. The script contains two Perl hashes with software and module download paths. Please comment out any pre-installed software/modules (as shown in the screenshot below for `YAML` and `cmake`) and update the path for them manually in `SomaticCallerConfig.pm`.

```
my %perl_dependency_hash = (
    Text_SNP => "http://search.cpan.org/CPAN/authors/id/T/TP/TPEDERSE/Text-NSP-1.25.tar.gz",
    Stat_Des => "http://search.cpan.org/CPAN/authors/id/C/CO/COLINK/Statistics-Descriptive-2.6.tar.gz",
    BioPerl => "http://bioperl.org/DIST/old_releases/bioperl-1.2.3.tar.gz",
    File_Grep => "http://search.cpan.org/CPAN/authors/id/M/MN/MNEYLON/File-Grep-0.02.tar.gz",
    #YAML => "http://search.cpan.org/CPAN/authors/id/M/MS/MSTROUT/YAML-0.84.tar.gz",
    DBI_MySQL => "http://search.cpan.org/CPAN/authors/id/T/TM/TMTM/Class-DBI-mysql-1.00.tar.gz",
    DBD_MySQL => "http://search.cpan.org/CPAN/authors/id/C/CA/CAPTTOFU/DBD-mysql-3.0002.tar.gz",
    Ensembl =>  "http://www.ensembl.org/cvsdownloads/ensembl-71.tar.gz",
    Ensembl_Variation => "http://www.ensembl.org/cvsdownloads/ensembl-variation-71.tar.gz"

);


my %tools_dependency_hash = (

    #cmake => "http://www.cmake.org/files/v2.8/cmake-2.8.10.2-Darwin64-universal.tar.gz",
    samtools => "http://sourceforge.net/projects/samtools/files/samtools/0.1.19/samtools-0.1.19.tar.bz2",
    vcftools => "http://sourceforge.net/projects/vcftools/files/vcftools_0.1.10.tar.gz",
    tabix => "http://sourceforge.net/projects/samtools/files/tabix/tabix-0.2.6.tar.bz2",
    bambino_jar => "https://cgwb.nci.nih.gov/cgi-bin/bambino?download_bambino_jar=bundle",
    varscan_jar => 'http://sourceforge.net/projects/varscan/files/VarScan.v2.3.5.jar',

);
```

Run the installation script as follows:

```
#download Cake
mkdir DIR_TO_TEST_CAKE
cd DIR_TO_TEST_CAKE
mkdir perl_lib
mkdir software_path
mkdir download_path
cd software_path
wget http://sourceforge.net/projects/cakesomatic/files/Cake_1.0.tar.gz
tar xfz Cake_1.0.tar.gz
```

Install required tools and configure Cake:

```
perl {DIR_TO_TEST_CAKE}/software_path/Cake/trunk/scripts/required_tools_installation.pl \
-perl_path               USER_PERL_PATH \ (example: /usr/bin/perl)
-perl_lib_path           {DIR_TO_TEST_CAKE}/perl_lib \
-download_path           {DIR_TO_TEST_CAKE}/download_path \
-tools_installation_path {DIR_TO_TEST_CAKE}/software_path \ (Make sure Cake is extracted in this directory)
```

-wget_location {PATH_TO_WGET} \ (If not available supply PATH_TO_CURL)
-curl_location {PATH_TO_CURL} \ (Mandatory if -wget not supplied)
-proxy_server_path Proxy_server_url \ (Optional - check using '$env | grep http_proxy')
-proxy_user_name Proxy_user_name \ (Optional)
-proxy_user_password Proxy_user_password \ (Optional)
-cake_installation_path {PATH_TO_CAKE_INSTALLATION_LOCATION} \ (Optional. Supply if different from 'tools_installation_path')
-config_file_path {PATH_TO_CAKE_CONFIGURATION_FILE} (Path to SomaticCallerConfig.pm file. Supply if different from the Cake installation location)

Check the software installation status in {DIR_TO_TEST_CAKE}/software_path/Software_Installation_Status.txt. If any software status indicates "Failed to Install" (See screenshot below), please follow the suggestion in the file. Alternatively, follow the manual installation guidance given below. Check the specified download path in the installation hash if any software installation fails.

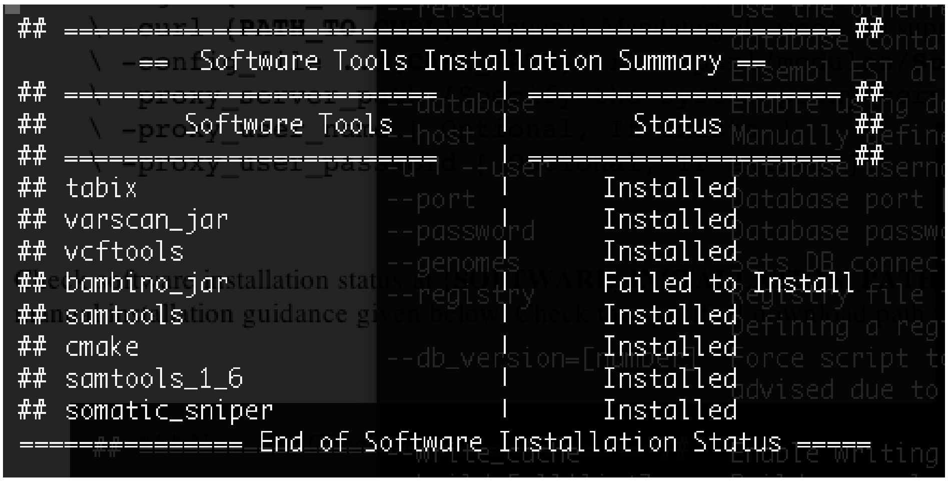

```
## ================================================ ##
     == Software Tools Installation Summary ==
## ======================  | ===================== ##
##     Software Tools      |        Status         ##
## ======================  | ===================== ##
## tabix                   |      Installed
## varscan_jar             |      Installed
## vcftools                |      Installed
## bambino_jar             |      Failed to Install
## samtools                |      Installed
## cmake                   |      Installed
## samtools_1_6            |      Installed
## somatic_sniper          |      Installed
=============== End of Software Installation Status =====
```

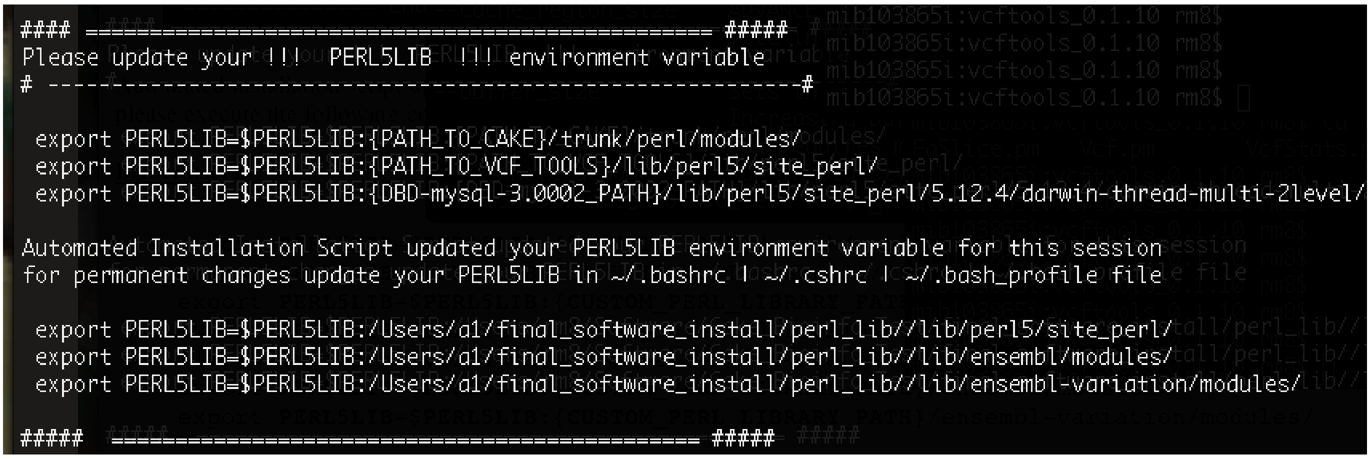

```
#### ============================================ #####
Please update your !!!  PERL5LIB  !!! environment variable
# ----------------------------------------------------------#

 export PERL5LIB=$PERL5LIB:{PATH_TO_CAKE}/trunk/perl/modules/
 export PERL5LIB=$PERL5LIB:{PATH_TO_VCF_TOOLS}/lib/perl5/site_perl/
 export PERL5LIB=$PERL5LIB:{DBD-mysql-3.0002_PATH}/lib/perl5/site_perl/5.12.4/darwin-thread-multi-2level/

Automated Installation Script updated your PERL5LIB environment variable for this session
for permanent changes update your PERL5LIB in ~/.bashrc | ~/.cshrc | ~/.bash_profile file

 export PERL5LIB=$PERL5LIB:/Users/a1/final_software_install/perl_lib//lib/perl5/site_perl/
 export PERL5LIB=$PERL5LIB:/Users/a1/final_software_install/perl_lib//lib/ensembl/modules/
 export PERL5LIB=$PERL5LIB:/Users/a1/final_software_install/perl_lib//lib/ensembl-variation/modules/

#####  ============================================ #####
```

The automated installation script updates the PERL5LIB environment variable for successfully installed Perl modules and software. Users are only required to update PERL5LIB for Cake modules. However, these changes are valid only for the current session. To update your environment variable permanently, please follow these instructions:

Bash users Add the following lines to your ~/.bash_profile or ~/.bashrc file (Please remember to change the paths in curly brackets for the locations in your system):

```
export PERL5LIB=$PERL5LIB:{PATH_TO_CAKE}/trunk/perl/modules/
export PERL5LIB=$PERL5LIB:{PATH_TO_VCFTOOLS_DIR}/lib/perl5/site_perl/
export PERL5LIB=$PERL5LIB:{CUSTOM_PERL_LIBRARY_PATH}/lib/perl5/site_perl/
```

```
export PERL5LIB=$PERL5LIB:{DBD-mysql-3.0002_PATH}/lib/perl5/site_perl/\
5.12.4/darwin-thread-multi-2level/
export PERL5LIB=$PERL5LIB:{CUSTOM_PERL_LIBRARY_PATH}/ensembl/modules/
export PERL5LIB=$PERL5LIB:{CUSTOM_PERL_LIBRARY_PATH}/ensembl-variation/modules/
```

C shell users Add the following lines to your ~/.cshrc file (Please remember to change the paths in curly brackets for the locations in your system):

```
setenv PERL5LIB $PERL5LIB:{PATH_TO_CAKE}/trunk/perl/modules/
setenv PERL5LIB $PERL5LIB:{PATH_TO_VCFTOOLS_DIR}/lib/perl5/site_perl/
setenv PERL5LIB $PERL5LIB:{CUSTOM_PERL_LIBRARY_PATH}/lib/perl5/site_perl/
setenv PERL5LIB $PERL5LIB:{DBD-mysql-3.0002_PATH}/lib/perl5/site_perl/\
5.12.4/darwin-thread-multi-2level/
setenv PERL5LIB $PERL5LIB:{CUSTOM_PERL_LIBRARY_PATH}/ensembl/modules/
setenv PERL5LIB $PERL5LIB:{CUSTOM_PERL_LIBRARY_PATH}/ensembl-variation/modules/
```

3.2.2 Manual installation

In case of a failure in the automated installation using the installation script, please follow this manual installation guideline. The tested versions for these tools are indicated.

Generic installation routine

```
download required software source code (skip if there is already a local copy)
tar xfz tool-name.version.tar.gz
cd tool-name.version
make
```

Additional manual installation instructions are indicated where necessary. Please remember to change {DOWNLOAD_PATH} for the real directory where you downloaded these tools.

3.2.3 samtools

Installation

```
wget http://sourceforge.net/projects/samtools/files/samtools/0.1.18/\
samtools-0.1.18.tar.bz2
tar -xjf samtools-0.1.18.tar.bz2
cd samtools-0.1.18
make
```

Variable to change in SomaticCallerConfig.pm:

```
SAM_TOOLS_BIN => {DOWNLOAD_PATH}/samtools-0.1.18/samtools
BCF_TOOLS_BIN => {DOWNLOAD_PATH}/samtools-0.1.18/bcftools/bcftools
```

3.2.4 tabix

Installation

```
wget http://sourceforge.net/projects/samtools/files/tabix/tabix-0.2.6.tar.bz
tar -xjf tabix-0.2.6.tar.bz2
```

Variable to change in SomaticCallerConfig.pm:

```
TABIX => {DOWNLOAD_PATH}/tabix-0.2.6/tabix
BGZIP => {DOWNLOAD_PATH}/tabix-0.2.6/bgzip
```

3.2.5 vcftools

Installation

```
wget http://sourceforge.net/projects/vcftools/files/vcftools_0.1.10.tar.gz
tar -xzf vcftools_0.1.10.tar.gz
cd vcftools_0.1.10
make
```

Update your PERL5LIB path

```
export PERL5LIB=$PERL5LIB:{DOWNLOAD_PATH}/lib/perl5/site_perl/ (Bash)
setenv PERL5LIB $PERL5LIB:{DOWNLOAD_PATH}/lib/perl5/site_perl/ (C shell)
```

Variable to change in SomaticCallerConfig.pm:

```
VCF_TOOLS_PATH => {DOWNLOAD_PATH}/bin/
```

3.2.6 Varscan 2

No installation is necessary. Only download the executable jar file.

```
wget http://sourceforge.net/projects/varscan/files/VarScan.v2.3.5.jar
```

Variable to change in SomaticCallerConfig.pm:

```
VARSCAN_JAR => {DOWNLOAD_PATH}/VarScan.v2.3.5.jar
```

3.2.7 Bambino

No installation is necessary. Only download the executable jar file.

```
wget_path -O bambino_bundle_1.03.jar https://cgwb.nci.nih.gov/cgi-bin/\
bambino?download_bambino_jar=bundle
```

Variable to change in SomaticCallerConfig.pm:

```
BAMBINO_JAR => {DOWNLOAD_PATH}/bambino_bundle_1.03.jar
```

3.2.8 cmake (Prerequisite for SomaticSniper, version $\geq$ 2.8)

Installation

```
wget http://www.cmake.org/files/v2.8/cmake-2.8.10.2.tar.gz
tar xzf cmake-2.8.10.2.tar.gz
cd cmake-2.8.10.2
./bootstrap --prefix={CURRENT_DIR}
make
make install
```

3.2.9 samtools_0.1.6 (Prerequisite for SomaticSniper)

Installation

```
wget http://sourceforge.net/projects/samtools/files/samtools/0.1.6/samtools-0.1.6.tar.bz2
tar -xjf samtools-0.1.6.tar.bz2
cd samtools-0.1.16
make
export SAM_TOOLS_ROOT={DOWNLOAD_PATH}/samtools (Bash)
setenv SAM_TOOLS_ROOT {DOWNLOAD_PATH}/samtools (C shell)
```

3.2.10 SomaticSniper

Prerequisites: cmake and samtools_0.1.6 (Must be intalled before installing SomaticSniper)

Installation

Linux distributions

```
wget -q -O - https://apt.genome.wustl.edu/ubuntu/files/install.sh | sudo bash
sudo gmt install somatic-sniper1.0.2
```

For more details: http://gmt.genome.wustl.edu/somatic-sniper/1.0.2/install.html

Source code installation

```
git clone --recursive git://github.com/genome/somatic-sniper.git
cd somatic-sniper
cmake {DOWNLOAD_PATH}
make
```

Variable to change in `SomaticCallerConfig.pm`:

```
SOMATICSNIPER => ../{DOWNLOAD_PATH}/bin/bam-somaticsniper
```

3.3 Perl modules

The required Perl modules and installation instructions, are given below. If you have run the `required_tools_installation.pl` script, you might have installed all of these already. If for any reason the installation of any of these modules fails, or for manual installation of all these libraries, detailed instructions are provided. After downloading from the specified source you can do (Please remember to change `{CUSTOM_PERL_LIB_PATH}` for the directory where you want to install these modules):

```
tar xvfz package-name.version.tar.gz
cd package-name.version
perl Makefile.PL PREFIX={CUSTOM_PERL_LIB_PATH}
make
make test
make install
```

After downloading all of these libraries (whether in manual or automatic installation), please make sure their paths are added to your `PERL5LIB` variable.

3.3.1 DBI

```
wget http://search.cpan.org/CPAN/authors/id/T/TM/TMTM/Class-DBI-mysql-1.00.tar.gz
tar xfz Class-DBI-mysql-1.00.tar.gz
cd Class-DBI-mysql-1.00
perl Makefile.PL PREFIX={CUSTOM_PERL_LIB_PATH}
make
make test
make install
export PERL5LIB=$PERL5LIB:{CUSTOM_PERL_LIB_PATH}
```

Please add `{CUSTOM_PERL_LIB_PATH}/lib/perl5/site_perl/` to your `PERL5LIB` variable.

3.3.2 DBD::MySQL

```
wget http://search.cpan.org/CPAN/authors/id/C/CA/CAPTTOFU/DBD-mysql-3.0002.tar.gz
tar xfz DBD-mysql-3.0002.tar.gz
cd DBD-mysql-3.0002
perl Makefile.PL PREFIX={CUSTOM_PERL_LIB_PATH}
make
make test
make install
export PERL5LIB=$PERL5LIB:{CUSTOM_PERL_LIB_PATH}
```

Note:

This version of DBD::MySQL has been tested on a MacBook Pro with a 64-bit processor (Intel Core 2 Duo), running Perl v5.12.4 and MySQL Server 5.5. The following environment variables were set:

```
export DYLD_LIBRARY_PATH=/usr/local/mysql/lib/:$DYLD_LIBRARY_PATH
export VERSIONER_PERL_PREFER_32_BIT=no;
```

You might need a different version of DBD::MySQL depending on your Perl and MySQL binaries.

3.3.3 YAML

```
wget http://search.cpan.org/CPAN/authors/id/M/MS/MSTROUT/YAML-0.84.tar.gz
tar xfz YAML-0.84.tar.gz
cd YAML-0.84
perl Makefile.PL PREFIX={CUSTOM_PERL_LIB_PATH}
make
make test
make install
export PERL5LIB=$PERL5LIB:{CUSTOM_PERL_LIB_PATH}
```

3.3.4 File::Grep

```
wget http://search.cpan.org/CPAN/authors/id/M/MN/MNEYLON/File-Grep-0.02.tar.gz
tar xfz File-Grep-0.02.tar.gz
cd File-Grep-0.02
perl Makefile.PL PREFIX={CUSTOM_PERL_LIB_PATH}
make
make test
make install
export PERL5LIB=$PERL5LIB:{CUSTOM_PERL_LIB_PATH}
```

3.3.5 Statistics::Descriptive

```
wget http://search.cpan.org/CPAN/authors/id/C/CO/COLINK/Statistics-Descriptive-2.6.tar.gz
tar xfz Statistics-Descriptive-2.6.tar.gz
cd Statistics-Descriptive-2.6
perl Makefile.PL PREFIX={CUSTOM_PERL_LIB_PATH}
make
make test
make install
export PERL5LIB=$PERL5LIB:{CUSTOM_PERL_LIB_PATH}
```

3.3.6 Text::NSP

```
wget http://search.cpan.org/CPAN/authors/id/T/TP/TPEDERSE/Text-NSP-1.25.tar.gz
tar xfz Statistics-Descriptive-2.6.tar.gz
cd Statistics-Descriptive-2.6
perl Makefile.PL PREFIX={CUSTOM_PERL_LIB_PATH}
make
make test
make install
export PERL5LIB=$PERL5LIB:{CUSTOM_PERL_LIB_PATH}
```

3.4 BioPerl and Ensembl APIs

To make use of consequence predictions generated by Ensembl Variant Effect Predictor (VEP) software tools users also need to install BioPerl 1.2.3 and the Ensembl Core and Variation APIs. If you have run the `required_tools_installation.pl` Perl script, you should have installed these already. However, if you have not, you can install them very easily via Concurrent Versions System (CVS - http://directory.fsf.org/wiki/CVS) by following the instructions in the Ensembl Project's webpage (http://www.ensembl.org/info/docs/api/api_cvs.html).
Otherwise, you can download and install these packages manually in the same way as the Perl modules above. Detailed instructions on how to do this can be found in http://www.ensembl.org/info/docs/api/api_installation.html.

> *Note.* These Ensembl URLs will download version 71 of the Ensembl Core and Variation APIs, which work with the GRCh37 human assembly and the GRCm38 mouse assembly. Please make sure your sequencing reads have been aligned to these references, if they have not, you will need to install the appropriate release of the Ensembl Core and Variation APIs.

3.4.1 BioPerl

```
wget http://bioperl.org/DIST/old_releases/bioperl-1.2.3.tar.gz
tar xfz bioperl-1.2.3.tar.gz
cd bioperl-1.2.3
perl Makefile.PL PREFIX={CUSTOM_PERL_LIB_PATH}
make
make test
make install
export PERL5LIB=$PERL5LIB:{CUSTOM_PERL_LIB_PATH}
```

Note. You will need version 1.2.3, as newer versions are not compatible with the Ensembl APIs.

3.4.2 Ensembl Core API

```
wget -O {CUSTOM_PERL_LIB_PATH}/ensembl-71.tar.gz http://www.ensembl.org/cvsdownloads/\
ensembl-71.tar.gz
tar xfz ensembl-71.tar.gz
cd ensembl
export PERL5LIB=$PERL5LIB:{CUSTOM_PERL_LIB_PATH}/ensembl/modules
```

3.4.3 Ensembl Variation API

```
wget -O {CUSTOM_PERL_LIB_PATH}/ensembl-variation-71.tar.gz http://www.ensembl.org/cvsdownloads/\
ensembl-variation-71.tar.gz
tar xfz ensembl-variation-71.tar.gz
cd ensembl-variation
export PERL5LIB=$PERL5LIB:{CUSTOM_PERL_LIB_PATH}/ensembl-variation/modules
```

3.4.4 Ensembl Tools

```
wget -O {CUSTOM_PERL_LIB_PATH}/ensembl-tools-71.tar.gz http://www.ensembl.org/cvsdownloads/\
ensembl-tools-71.tar.gz
tar xfz ensembl-tools-71.tar.gz
cd ensembl-tools
```

Variable to change in `SomaticCallerConfig.pm`:

```
ENSEMBL_VARIANT_EFFECT_PREDICTOR => {CUSTOM_PERL_LIB_PATH}/ensembl-tools/scripts/\
variant_effect_predictor/variant_effect_predictor.pl
```

3.5 Update your `PERL5LIB` environment variable

Please add the following paths to your `PERL5LIB` environment variable, if you haven't done so already (To add these paths permanently to your environment variables, you can add the following lines to your `~/.bash_profile`, `~/.bashrc` or `~/.cshrc` files. Remember to change the paths in the curly brackets for the paths in which you installed these tools!):

For Bash users

```
export PERL5LIB=$PERL5LIB:{CAKE_PATH}/trunk/perl/modules/
export PERL5LIB=$PERL5LIB:{VCFTOOLS_DOWNLOAD_PATH}/lib/perl5/site_perl/
export PERL5LIB=$PERL5LIB:{CUSTOM_PERL_LIB_PATH}/lib/perl5/site_perl/
export PERL5LIB=$PERL5LIB:{CUSTOM_PERL_LIB_PATH}/lib/perl5/site_perl/5.12.4/\
darwin-thread-multi-2level/
export PERL5LIB=$PERL5LIB:{CUSTOM_PERL_LIB_PATH}/ensembl/modules/
export PERL5LIB=$PERL5LIB:{CUSTOM_PERL_LIB_PATH}/ensembl-variation/modules/
```

For C shell users

```
setenv PERL5LIB $PERL5LIB:{CAKE_PATH}/trunk/perl/modules/
setenv PERL5LIB $PERL5LIB:{VCFTOOLS_DOWNLOAD_PATH}/lib/perl5/site_perl/
setenv PERL5LIB $PERL5LIB:{CUSTOM_PERL_LIB_PATH}/lib/perl5/site_perl/
setenv PERL5LIB $PERL5LIB:{CUSTOM_PERL_LIB_PATH}/lib/perl5/site_perl/5.12.4/\
darwin-thread-multi-2level/
setenv PERL5LIB $PERL5LIB:{CUSTOM_PERL_LIB_PATH}/ensembl/modules/
setenv PERL5LIB $PERL5LIB:{CUSTOM_PERL_LIB_PATH}/ensembl-variation/modules/
```

3.6 Additional files

Cake uses an array of variant filtering modules (e.g. common SNPs filter or the removal of non-exonic variants) to reduce the number of potential somatic variants. These filtering modules are optional and can be run independently. Users need to provide relevant files (e.g. a list of known SNPs or an exonic region file in the previously mentioned examples) for the filtering module to work effectively. In the following sections we will discuss details about the format and possible sources for these additional files. Please check the tables in **Section 4.6** for more details on how to use the filters. The paths to these files need to be specified in the `SomaticCallerConfig.pm` file.

3.6.1 Necessary files

A reference genome in FASTA format. You can download these from the Genome Reference Consortium's webpage
Human - Hyperlink to human reference fasta file, GRCh37
Mouse - Hyperlink to mouse reference fasta file, GRCm38
Please make sure that the reference you use is the same that your sequencing reads have been aligned to (i.e. BAM files). You will also need to index your FASTA file, and you can do this with Samtools:

```
{SAMTOOLS_PATH}/samtools faidx reference.fa
```

Please add this path to the `REFERENCE_FASTA => [HUMAN|MOUSE]` variable as appropriate.

3.6.2 Optional files

A list of exonic coordinates, if you are working with exomes (if the `EXONIC_FILTER` flag is set to `TRUE`). You can use the provided files or you can provide your own set of targeted regions. The required format for these is a list of the form

`chr<tab>start position<tab>end position` (1-based coordinates)

Please add this path to the `EXON_COORDINATES_FILE => [HUMAN|MOUSE]` variable as appropriate.

For example the generic human exonic regions can be downloaded as

```
mysql -u anonymous -h ensembldb.ensembl.org -P 5306 -D homo_sapiens_core_61_37f -A
-e 'select S.stable_id,R.name,E.seq_region_start,E.seq_region_end,E.seq_region_strand from exon as E,seq_region as R,ex
on_stable_id as S where R.seq_region_id=E.seq_region_id and S.exon_id=E.exon_id'
+-----------------+------+------------------+----------------+-------------------+
| stable_id       | name | seq_region_start | seq_region_end | seq_region_strand |
+-----------------+------+------------------+----------------+-------------------+
| ENSE00002029850 | 5    |         94120533 |       94120602 |                -1 |
| ENSE00002069321 | 4    |         17835922 |       17836146 |                 1 |
| ENSE00002048418 | 5    |        123731640 |      123731794 |                -1 |
| ENSE00001815244 | 6    |         13711167 |       13711796 |                -1 |
| ENSE00001363151 | 2    |          1507720 |        1507851 |                 1 |
| ENSE00001737796 | 1    |         40537122 |       40537924 |                 1 |
| ENSE00001800436 | 2    |        165208630 |      165208733 |                -1 |
| ENSE00001255746 | 10   |         93683822 |       93683847 |                 1 |
| ENSE00001844789 | 14   |         74523609 |       74523683 |                 1 |
| ENSE00002137765 | 8    |         17541844 |       17542051 |                -1 |
(...)
```

Note. Please make sure that these coordinates are 1-based!

A list of positions to discard in the common variation filtering step (if the `SNP_FILTER` flag is set to `TRUE`). If you are working with mouse data, you might want to discard germline variants found in the genomes of 17 mouse strains (Thomas M. Keane et al, Nature 2011). A list of SNPs can be downloaded from the Sanger FTP website. Alternatively, if you are working with human data, you might want to discard the SNPs found in the dbSNP database (Human SNPs file) or the 1000 Genomes Project (1000 Genomes SNPs website). The required format for these is a list of the form

```
chr<tab>position
```

Please add this path to the `GENOMES => [HUMAN|MOUSE] => [1000_GENOME_SNP|17_MOUSE_GENOME_SNP]` variable as appropriate. You can add all the common variant files that you need by creating additional variables, and you can modify the variable names. See the example snapshot from `SomaticCallerConfig.pm`

```
GENOMES =>
{
    MOUSE =>
    {
        '17_MOUSE_GENOME' => 'PATH_to_17_Mouse_Genome_SNP_File',

    },

    HUMAN =>
    {
        '1000_GENOME_SNP' =>'PATH_to_1000_Genome_Genome_SNP_File',
        'UK10K_SNP' =>'PATH_to_UK10K_Genome_Genome_SNP_File',
    },
},
```

A list of known indel positions to discard in the indel filtering step (if the `INDEL_FILTER` flag is set to `TRUE`). If you are working with mouse data, you might find the list of indels annotated in the genomes of 17 mouse strains useful (Thomas M. Keane et al, Nature 2011). A list of these can be downloaded from here. The required format for these is the same as the one specified for exonic coordinates.

Please add this path to the `INDEL_FILE => [HUMAN|MOUSE]` variable as appropriate.

A list of COSMIC-annotated regions in BED format (if `COSMIC_ANNOTATION_FLAG` is set to `TRUE`). If you are working with human data, you might want to mark up mutations that have been seen previously in other cancers and are stored in the COSMIC, or any other database. If so, please provide a file with a list of positions in the following format

```
chr<tab>start position<tab>end position
```

Please add this path to the `COSMIC_MUTATIONS_FILE` variable.

3.6.3 Ingredients check

Using Cake's configuration checker tool you can check whether the software tools and other relevant files (e.g reference fasta file, exonic cordinate file, etc.) paths are correctly configured in `SomaticCallerConfig.pm` file:

```
perl {CAKE_PATH}/trunk/scripts/pipeline_config_checker.pl \
-species mouse \
-callers mpileup,varscan,bambino,somaticsniper \
-o {USER_SPECIFIED_OUTPUT_DIR}
```

4 Running Cake

Congratulations! You have now installed and configured all the software prerequisites for running Cake. You can now proceed to run it.

4.1 Sample information file

This is a simple comma- or tab-separated text file specifying your tumour and normal BAM files (see following example). Depending on the selected run mode and available architecture (**Section 4.2**) it can run mutiple samples in either sequential or parallel mode.

SampleID	NormalID	Cohort	PatientID	Type	TumorBAM	NormalBAM
Tumor1	Normal1	Leukaemia	P1	Tumour	PATH To Tumour 1 BAM File	PATH To Normal 1 BAM File
Tumor2	Normal2	Leukaemia	Unknown	Tumour	PATH To Tumour 2 BAM File	PATH To Normal 2 BAM File

4.2 Run modes

Cake can be run either in standalone or distributed mode. So far Cake only supports the LSF job managament system in the distributed mode. Depending on your local system type, please modify the `SYSTEM_TYPE` and `CLUSTER_TYPE` fields in `SomaticCallerConfig.pm`. Check the table in **Section 4.4** for more details about these parameters.

4.3 Run Cake

The Cake somatic variant pipeline comprises two major components:

1. Variant calling pipeline
2. Variant filtering pipeline

The variant calling module detects somatic variants using various publicly available variant-calling tools (e.g. Bambino, samtools mpileup, VarScan 2 and SomaticSniper). The variant filtering section of the pipeline combines somatic variants from mutiple callers to produce a reliable set of somatic variants, which are processed through variant-filtering modules to increase the sensitivity and specificity of the pipeline (See Supplementary Figure 1). These two components of Cake can be run independently. A customisable list of parameters allows the user to set different filtering strategies as well as their stringency levels. These parameters have default values and can be modified within the `SomaticCallerConfig.pm` file. The tables in this section contain the full list of these parameters, their description and default values.

```
perl {CAKE_PATH}/trunk/scripts/run_cake_somatic_pipeline.pl \
-s {PATH_TO_SAMPLE_INFORMATION_FILE} \
-species [mouse|human] \
-callers mpileup,varscan,bambino,somaticsniper \
-separator [,|\t] \
-o {PATH_TO_USER_SPECIFIED_OUTPUT_DIR} \
-mode [FULL|CALLING|FILTERING]
```

An explanation of these command-line parameters is found in the following table.

Flag	Description
`-s`	Path to the sample information file (**Section 4.1**). An example of an accepted format can be found in `{CAKE_PATH}/Cake/example/sample_specification.csv`. The fields in this file can be comma- or tab-separated, which then has to be specified in the `-separator` parameter.
`-species`	The species studied by the present study. *Allowed options*: `human` or `mouse`.
`-callers`	The algorithms to be run by the pipeline. *Allowed options*: Any combination of algorithms for which the paths have been specified in the `SomaticCallerConfig.pm` file, separated by a comma.
`-separator`	The field separator in the sample specification file. *Allowed options*: `,` or `\t`.
`-o`	Path of the output directory, where all files created by the pipeline will be stored.
`-mode`	The mode in which the pipeline will be run. *Allowed options*: `CALLING`, in which only the variant calling and merging will be run. `FILTERING`, in which only the post-processing phase will be run, if you have previously called with a different algorithm. `FULL`, in which both phases will be run.

The variant calling component is more computationally expensive and time consuming compared to the variant filtering module, which finishes fairly quickly (1-2 hours per exome on a 2.5GHz CPU with 4 GB memory).

Therefore, the ideal running strategy would be

- Run the variant calling pipeline with a standard set of variant calling parameters allowing a large number of variants to pass through
- Make a backup copy of the VCF files generated by the different variant callers.
- Create multiple copies of the VCF files to try different filtering stringency and strategies.

4.4 General parameters

There are some general parameters that can be adjusted according to specific user requrements. The following table provides an explanation and the default value of these.
You can change their values in the `SomaticCallerConfig.pm` file.

Parameter	Description	Default value
	Computer architecture	
`SYSTEM_TYPE`	Type of computing architecture for run mode. Allowed values: `CLUSTER`, `STANDALONE`	`CLUSTER`
`CLUSTER_TYPE`	Type of computer cluster. Allowed values: `LSF` (Only implemented if `SYSTEM_TYPE` is set to `CLUSTER`).	`LSF`
`MAX_NUM_SAMPLES_RUNNING`	Number of samples to be run at the same time (Only implemented if `SYSTEM_TYPE` is set to `CLUSTER`).	`10`
`GROUP`	LSF group ID (Only implemented if `SYSTEM_TYPE` is set to `CLUSTER`).	*none*
`BAMBINO_QUEUE`<br>`MPILEUP_QUEUE`<br>`VARSCAN_QUEUE`<br>`SOMATICSNIPER_QUEUE`<br>`POST_PRO_QUEUE`	The queue to which jobs will be submitted for each of the implemented variant calling algorithms and the variant filtering steps. Ignore the parameter if it's not relevant to the current study. (Only implemented if `SYSTEM_TYPE` is set to `CLUSTER`).	`normal`
`TMP_DIR`	Directory to which temporary files will be written	`/tmp/`
	Sequencing data type	
`SEQUENCING_DATA_TYPE`	Type of NGS data to analyse. Allowed values: `exome`, `genome`	`exome`

4.5 Variant calling parameters

To run Cake in variant calling mode only, you can try:

```
perl {CAKE_PATH}/trunk/scripts/run_cake_somatic_pipeline.pl \
-s {PATH_TO_SAMPLE_INFORMATION_FILE} \
-species [mouse|human] \
-callers mpileup,varscan,bambino,somaticsniper \
-separator [,|\t] \
-o {PATH_TO_USER_SPECIFIED_OUTPUT_DIR} \
-mode CALLING
```

You can change the values of the following variant calling parameters in the `SomaticCallerConfig.pm` file:

Parameter	Description	Default value
	Generic variant calling parameters	
`MIN_MAPPING_QUALITY`	Minimum mapping quality. Reads with a mapping quality lower than the specified value will be discarded.	`15`
`MIN_NUCLEOTIDE_QUALITY`	Minimum base quality. Variants called in a read with a lower base quality than the specified value will be discarded.	`10`

MIN_COVERAGE	Minimum variant depth. Variants called with a combined tumour + germline depth less than the specified value will be discarded.	4
MIN_BASE_POSITION_FREQUENCY	Filter for the proportion of reads that show a variant in the first two thirds of its length. If the proportion of reads that present this variant within their first two thirds is less than the specified value, then the variant is discarded.	0.6
	Bambino-specific parameters	
MIN_FLANKING_QUALITY	Minimum quality of flanking sequences	15
MIN_ALT_ALLELE_COUNT	Minimum number of reads displaying the alternative allele	2
MMF_MAX_HQ_MISMATCHES	Minimum number of high-quality mismatches in a read	5
MMF_MIN_HQ_THRESHOLD	Minimum read quality. Reads with a quality less than the specified value will be discarded.	15
MMF_MAX_LQ_MISMATCHES	Minimum number of low-quality mismatches in a read	6
UNIQUE_FILTER_COVERAGE	Minimum number of unique reads covering the variant	2
	Samtools mpileup-specific parameters	
BWA_DOWNGRADE_COFF	Coefficient for downgrading mapping quality for reads containing excessive mismatches	50
NO_OF_READS_TO_ CONSIDER_REALIGNMENT	Number of reads to initiate realignment	3
FREQ_OF_READS	Frequency of supporting reads to initiate realignment	0.0002
MPILEUP_QUALITY_THRESHOLD	Quality score	10
	SomaticSniper-specific parameters	
SOMATIC_QUALITY	Quality of the variant	15
	CaVEMan-specific parameters	
NO_OF_BASES_TO_INCREMENT	Size of window (in bp) into which CaVEMan divides jobs	250000

4.6 Variant filtering flags and parameters

If you have independently run a variant-calling algorithm and would like to run only the variant-filtering steps, you can do as follows:

- Add the caller information to `{CAKE_PATH}/trunk/scripts/somatic_post_processing_caller.pl` (Detailed instructions are in the file header)
- Place the output VCF file in the output directory designated for that particular sample.

To run Cake in variant-filtering-only mode, you can do:

```
perl {CAKE_PATH}/trunk/scripts/run_cake_somatic_pipeline.pl \
-s {PATH_TO_SAMPLE_INFORMATION_FILE} \
-species [mouse|human] \
-callers mpileup,varscan,bambino,somaticsniper \
-separator [,|\t] \
-o {PATH_TO_USER_SPECIFIED_OUTPUT_DIR} \
-mode FILTERING
```

The different filters you can run as part of the filtering phase are specified in the `SomaticCallerConfig.pm` file, and you can set them as `TRUE` (the filter will be implemented) or `FALSE` (it will be skipped):

Parameter	Description	Default value
EXONIC_FILTER	Whether or not to retain only variants that fall in exonic regions. If `TRUE`, `EXON_COORDINATES_FILE => [HUMAN|MOUSE]` must be specified.	TRUE
SNP_FILTER	Whether or not to filter variants that overlap a defined set (for example, common variants in the population). If `TRUE`, `GENOMES => [HUMAN|MOUSE] => [DATASET NAME]` must be specified.	TRUE

INDEL_FILTER	Whether or not to discard variants that fall within known indels, or the specified genomic intervals. If TRUE, INDEL_FILE => [HUMAN|MOUSE] must be specified.	TRUE
GERMLINE_FILTER	Whether or not to discard variants that are found in the matched germline sample.	TRUE
BASE_POS_FILTER	On a per-read basis, whether or not to discard variants that fall within the last third position-wise (Which generally have much lower quality)	TRUE
SOMATIC_COVERAGE_FILTER	Whether to require that variants have a minimum depth in both tumour and germline samples. If TRUE, TUMOR_MIN_DEPTH and NORMAL_MIN_DEPTH must be specified.	TRUE
ANNOTATE_CONSEQUENCE	Whether to annotate the predicted consequences of the set of identified variants.	FALSE
FILTER_CONSEQUENCE	Whether to retain only a certain set of consequences. If TRUE, variants specified in CONSEQUENCE_TO_FILTER will be discarded.	FALSE
ALLELE_RATIO_FILTER	Per variant, whether or not to apply a minimum ratio for the proportion of reads displaying it.	TRUE
COSMIC_ANNOTATION_FLAG	Whether or not to annotate the variants that have been seen before in the COSMIC dataset. If TRUE, COSMIC_MUTATIONS_FILE must be speficied.	TRUE

There are also some additional parameters that you can set in the same file:

Parameter	Description	Default value
Somatic coverage parameters		
TUMOR_MIN_DEPTH	Minimum variant depth in the tumour. Variants with a depth less than the specified value in the tumour alignment will be discarded if the SOMATIC_COVERAGE_FILTER flag is set to TRUE.	10
NORMAL_MIN_DEPTH	Minimum variant depth in the germline. Variants with a depth less than the specified value in the germline alignment will be discarded if the SOMATIC_COVERAGE_FILTER flag is set to TRUE.	10
General variant filtering parameters		
NO_VCF_TO_INTERSECT	Minimum number of algorithms to intersect calls, *position-based.* Positions that are called by less algorithms than the specified value will be discarded.	2
NO_GENOTYPE_TO_INTERSECT	Minimum number of algorithms to intersect calls, *genotype-based.* At least the specified number of algorithms have to agree on the called genotype for the variant to be mantained.	2
TUMOR_MIN_MINOR_ALLELE	Minimum proportion of reads in the tumour sample showing the alternative allele to call as heterozygous	0.1
TUMOR_MAX_MINOR_ALLELE	Maximum proportion of reads in the tumour sample showing the alternative allele to call as heterozygous	0.5
NORMAL_MIN_MINOR_ALLELE	Minimum proportion of reads in the germline sample showing the alternative allele to call as heterozygous	0.25
NORMAL_MAX_MINOR_ALLELE	Maximum proportion of reads in the germline sample showing the alternative allele to call as heterozygous	0.75
RATIO_FILTER_THRESHOLD	$(\frac{NVAR}{TVAR})$, where NVAR and TVAR refer to the proportion of reads displaying the variant allele in the germline and tumour samples, respectively. Only variants that are equal to or below the specified value will be retained.	0.1
ENSEMBL_DATABASE_VERSION	Version of the Ensembl release to be used in consequence prediction if the FILTER_CONSEQUENCE flag is set to TRUE.	71

`CONSEQUENCE_TO_FILTER`	A comma-separated list of the consequences to discard from the final output, if the `FILTER_CONSEQUENCE` flag is set to `TRUE`. Please note these must be the same terms that the Variant Effect Predictor (VEP) uses. Please see the VEP website for details.	All silent consequences. Please see script for details.
`EMPTY_CONSEQ_FILTER`	Boolean flag specifying if those variants for which the Ensembl VEP was unable to predict a consequence should be kept. If set to `FALSE`, empty-consequence variants will be removed.	`TRUE`

4.7 Output files

Once Cake has finished running, it will have generated several output files/directories in a user-defined output directory depending on the user-defined steps. Output files/directories will be organized according to the following directory structure.

OUTPUT_DIR

- ***Tumor1*** [*As specified in the first column of sample information file*]
  - ***Variant caller_1***
    - Variant caller specific output files
    - Converted VCF file
    - temp
  - ***Variant caller_2***
    - Variant caller specific output files
    - Converted VCF file
    - temp
  - ***……..***
  - ***Variant caller M*** [*M =number of variant caller used*]
  - ***Final_Output_Files***
  - ***Intermediate_Output_Files***
- ……
- ……
- ***TumorN***

For each sample (each row in the sample information file) there will be a directory designated to its output files. The final output file for a sample will be produced in the VCF format and can be found in the `Final_Output_Files` directory in the structure above. Cake generates intermediate VCF files after each variant-filtering module, adds a tag at the end and stores these files in the `Intermediate_Output_Files` directory. Caller-specific intermediate files are stored in a `/temp` directory inside in each caller directory.

Cake combines potential variants from multiple callers in a file named `SampleID_vs_NormalID.common_position.vcf`, with the various tags in the file name referring to the different filtering stages it has gone through.

A final output file that has been through all the variant-filtering steps will look like

```
SampleID_vs_NormalID.common_position.exonic_interval.known_snp.indels.germline\
.base_position.somatic_coverage.consequence_filtered.vcf
```

Filter (see **Section 4.6**)	Filter details	VCF tag
Common positions	Combines variant calls from multiple callers based on their genomic position and genotype match and stores them in a VCF file Controlled by `NO_VCF_TO_INTERSECT` and `NO_GENOTYPE_TO_INTERSECT`	`common_positions`
Exonic intervals	`EXONIC_FILTER` is set to `TRUE`. A test file can be found here	`exonic_interval`
Known positions	`SNP_FILTER` is set to `TRUE`. A test file can be foundhere	User-specified known positions file tags (as specified in config file) will appear in the tag.
Known indels	`INDEL_FILTER` is set to `TRUE`. A test file can be found here	`indels`
Germline	`GERMLINE_FILTER` is set to `TRUE`	`germline`
Base position	`BASE_POS_FILTER` is set to `TRUE`	`base_position`
Somatic coverage	`SOMATIC_COVERAGE_FILTER` is set to `TRUE`	`somatic_coverage`
Consequence	`FILTER_CONSEQUENCE` is set to `TRUE`	`consequence_filtered`
COSMIC annotation	`COSMIC_ANNOTATION_FLAG` is set to `TRUE`	`cosmic_annotation`

5 Example run

To run an example in a standalone environment, we have created a small test case. BAMs and other relevant files are distributed with Cake. Please make sure you successfully run the test case before you move on to your data. The files can be found in `{CAKE_PATH}/trunk/test_relevant_files/`. Or, please click here to download these files.

Download Cake

```
mkdir DIR_TO_TEST_CAKE
cd DIR_TO_TEST_CAKE
mkdir perl_lib
mkdir software_path
mkdir download_path
cd software_path
wget http://sourceforge.net/projects/cakesomatic/files/Cake_1.0.tar.gz
tar xfz Cake_1.0.tar.gz
```

Installation and configuration of required tools

```
perl {DIR_TO_TEST_CAKE}/software_path/Cake/trunk/scripts/required_tools_installation.pl \
-perl_path                USER_PERL_PATH \ (example: /usr/bin/perl)
-perl_lib_path            {DIR_TO_TEST_CAKE}/perl_lib \
-download_path            {DIR_TO_TEST_CAKE}/download_path \
-tools_installation_path  {DIR_TO_TEST_CAKE}/software_path \ (Make sure Cake is ex-
tracted in this directory)
-wget_location            {PATH_TO_WGET} \ (If not available supply PATH_TO_CURL)
-curl_location            {PATH_TO_CURL} \ (Mandatory if -wget not supplied)
-proxy_server_path        Proxy_server_url \ (Optional - check using '$env | grep http_proxy')
-proxy_user_name          Proxy_user_name \ (Optional)
-proxy_user_password      Proxy_user_password \ (Optional)
-cake_installation_path   {PATH_TO_CAKE_INSTALLATION_LOCATION} \ (Optional. If differ-
ent from 'tools_install_path')
```

-config_file_path {PATH_TO_CAKE_CONFIGURATION_FILE} (Path to SomaticCallerConfiguration.pm file. Supply if different from the Cake installation location)

This script will install all the necessary software and Perl modules required to run Cake. It also updates the SomaticCallerConfig.pm file with the Cake test run files and other relevant files. You have to change your PERL5LIB environment variable after successful completion of the automated installation (See **Section 3** for details).

Check Cake configuration

```
perl {DIR_TO_TEST_CAKE}/software_path/Cake/trunk/scripts/pipeline_config_checker.pl \
-s {DIR_TO_TEST_CAKE}/software_path/Cake/trunk/test_relevant_files/Sample_Information_File/\
Cake_Example_Run_SampleInformation_File.csv \
-species mouse \
-callers mpileup,varscan,bambino,somaticsniper \
-o {DIR_TO_TEST_CAKE}/software_path/Cake/trunk/test_relevant_files/ \
-separator ,
```

Run Cake

```
perl {DIR_TO_TEST_CAKE}/software_path/Cake/trunk/scripts/run_cake_somatic_pipeline.pl \
-s {DIR_TO_TEST_CAKE}/software_path/Cake/trunk/test_relevant_files/Sample_Information_File/\
Cake_Example_Run_SampleInformation_File.csv \
-species mouse \
-callers mpileup,varscan,bambino,somaticsniper \
-o {DIR_TO_TEST_CAKE}/software_path/Cake/trunk/test_relevant_files/ \
-separator , \
-mode FULL
```

The test set consists of two pairs of tumour/normal samples from a short strech of mouse chromosome 19. The test set is run with all four callers and the default filtering approach, and it will generate two directories, MouseT1 and MouseT2, inside the {USER_SPECIFIED_OUTPUT_FILE} directory. Inside each sample directory, the final output file can be found in the Final_Output_Files directory. The final output files should report four and eight somatic variants for MouseT1 and MouseT2 respectively.

Sample Cake output file:

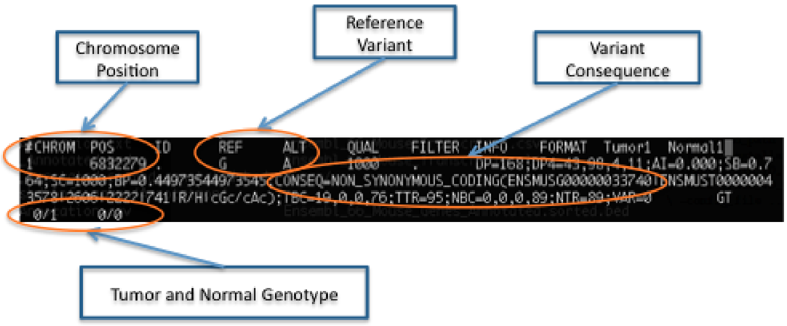

Supplement: Supplementary Data [file supp_btt371_20130604_user_manual_first_revision.pdf]
