# Supplementary material for: Cake: a bioinformatics pipeline for the integrated analysis of somatic variants in cancer genomes
Source: Bioinformatics. 2013 Jul 16;29(17):2208–10. doi: 10.1093/bioinformatics/btt371 (PMC3740632; doi:10.1093/bioinformatics/btt371)
Supplement: Supplementary Data [file supp_btt371_20130606_CAKE_Supp_Information.pdf]

Supplementary Figure 1

The Cake pipeline

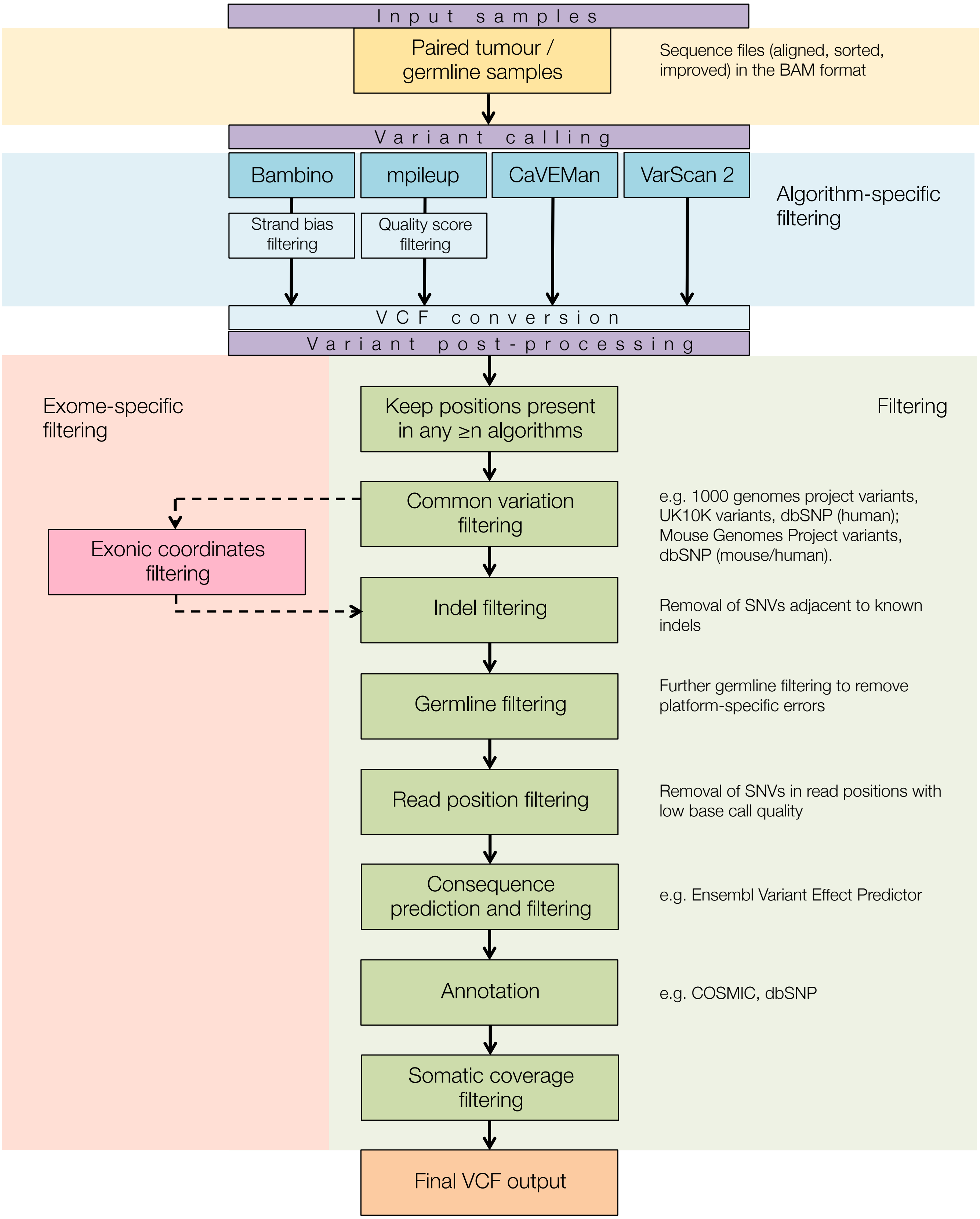

Supplementary Figure 2a

Variant intersection strategy

Somatic variant callers produce outputs in different formats, e.g. genotype (VCF) or read counts. For uniformity and better compatibility, Cake converts all outputs to VCF format. By default, variants identified by at least any 2 out of 4 callers and reporting the same genotypes are processed through variant filtering. In the example below, all algorithms have called the same genotype at Position 1 in both the tumour and the normal samples, and thus this variant will be considered for filtering using all intersection approaches (right side of the Figure 2a). Conversely, Position 4 is identified by 3 callers. Only two of them have called the same genotype. In this case the variant will passed the 'any out of 4' and 'at least 2 out of 4' callers strategies (Green and orange dotted rectangles).

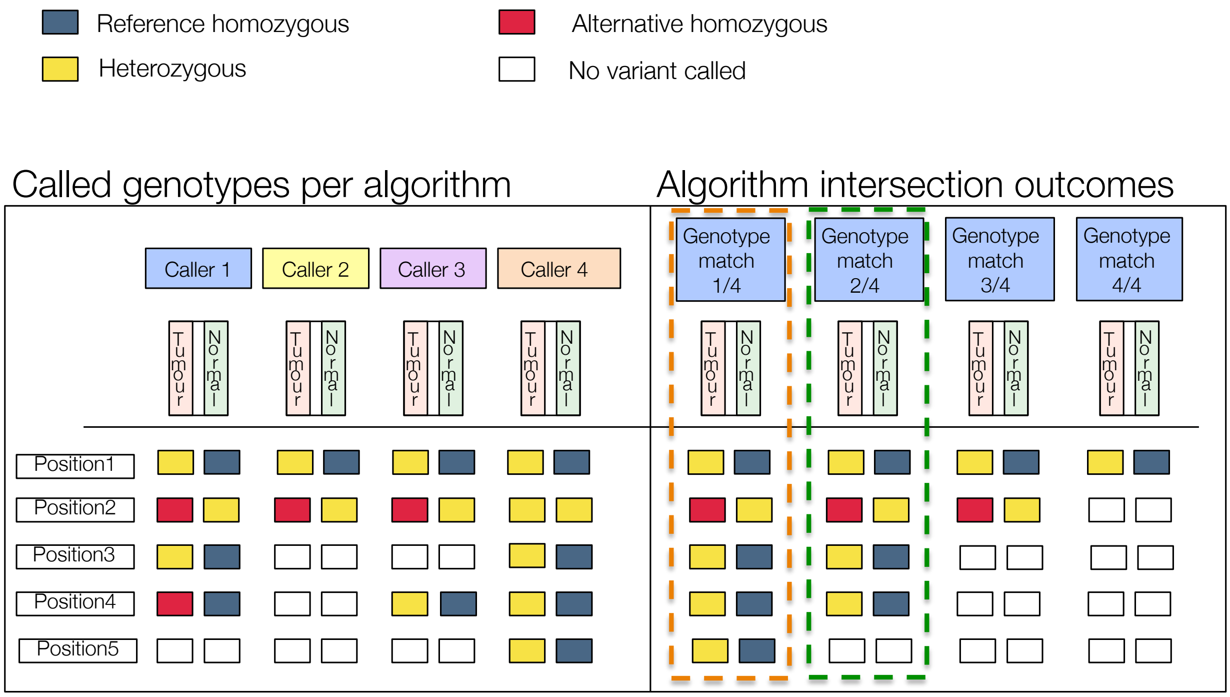

Figure 2a: Cake intersection strategy

Increased sensitivity

Through this flexible intersection approach, Cake seeks to improve the sensitivity as well as the specificity. For a variant to pass through the intersection stage, it has to be identified by at least any *n* (*n* = number of callers specified by the user in the configuration file) out of all (4 by default) somatic callers. Variants missed by one caller may be detected by others, contributing to higher sensitivity. Moreover, overlapping across multiple callers helps to control the false positive rate.

Supplementary Figure 2b

Variant intersection strategy

Best intersection strategy

Choosing the best overlapping strategy is a non-trivial problem considering the complex landscape of cancers. For example, variable mutation rates across different cancer types, combined with differences in sequencing technologies, make it difficult to generate a generic simulation data set.

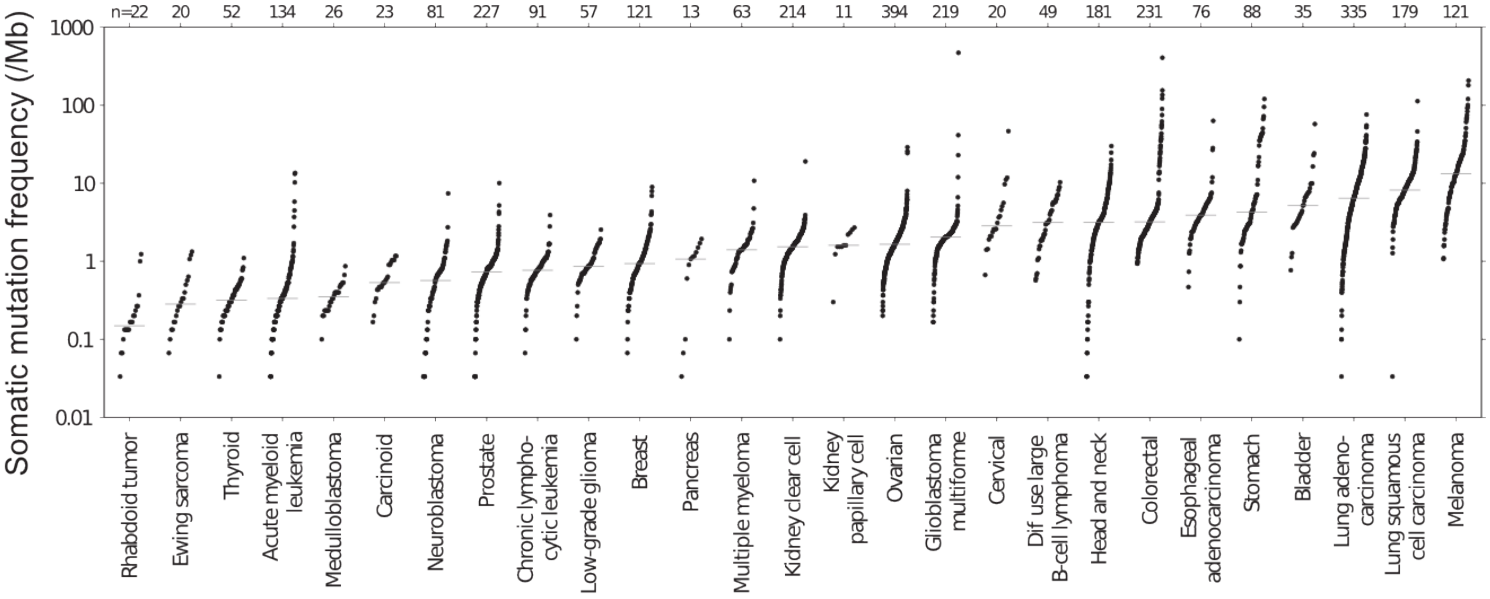

Figure 2b: Spectra of somatic mutations across cancer types [Taken from Lawrence *et al*. (2013) Nature, in press].

Validation capacity (the availability of large-scale validation technologies and resources) also restricts the number of mutations/genes that may be followed up. In Supplementary Table 3 we provide a guide to users of Cake to help them select the best overlapping strategy to deploy according to their data.

Supplementary Figure 3

Human hepatocellular carcinoma data

In their study, Guichard *et al.* (2012) validated 850 single-nucleotide somatic variants from 24 human hepatocellular carcinoma tumour/normal exome pairs. At eight of these sites, we were unable to find read coverage following re-alignment of the data. These positions were excluded from downstream analysis. This left a control set of 842 somatic SNV positions. Using the Cake merge approach (≥ any 2 out of 4 callers), we identified 812 of these positions. The Venn diagram below shows the breakdown of these calls by caller and the overlapping calls.

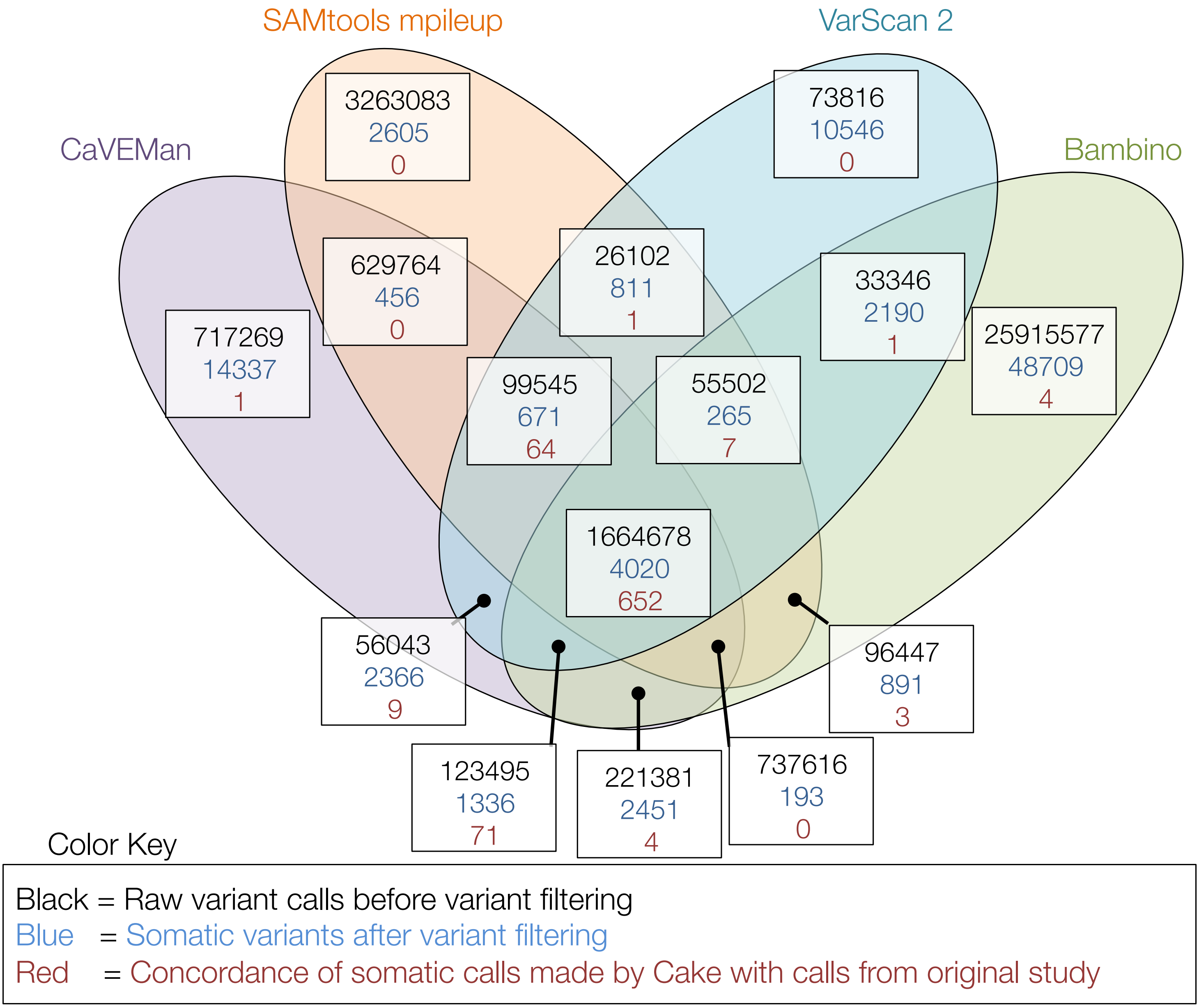

Supplementary Figure 4

Human breast cancer data

Stephens *et al.* (2012) validated 264 somatic mutations from two breast cancer tumour/germline exome pairs. Here we show somatic variant calls made by the algorithms in the Cake pipeline. Using an intersection of calls made by ≥ any 2 out of 4 callers, followed by variant filtering, we identified 254 of the 264 validated positions.

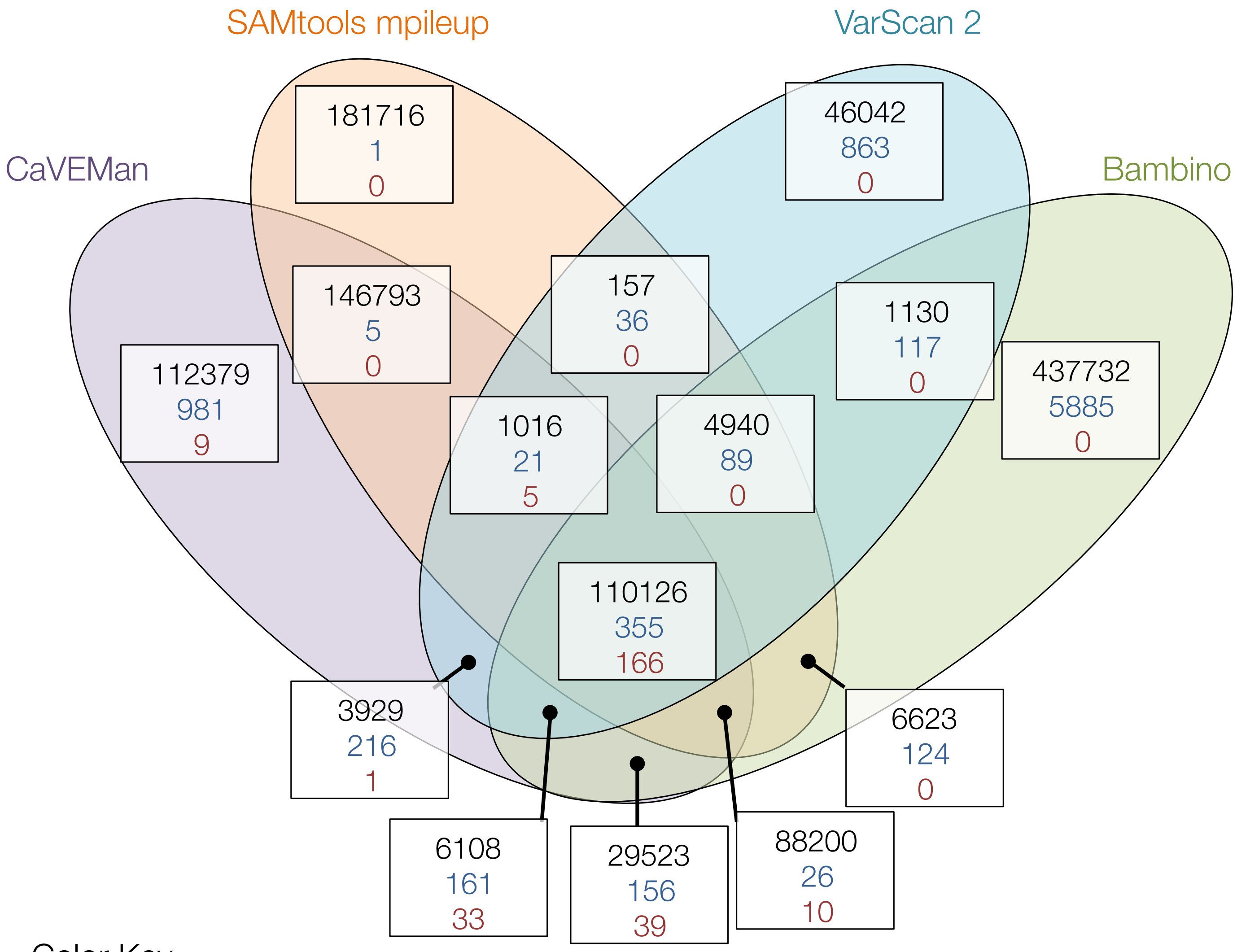

Color Key

Black = Raw variant calls before variant filtering
Blue = Somatic variants after variant filtering
Red = Concordance of somatic calls made by Cake with calls from original study

Supplementary Figure 5

Human breast cancer data: validation

To assess the sensitivity and specificity of the Cake merge approach (≥ any 2 out of 4 callers), we sent 400 predicted somatic variants for independent validation in the Sequenom MassArray platform. The Venn diagram below shows the distribution and breakdown of these variants (novel as well as those validated in the original study). Variants that failed at any stage during the validation and for which we could not determine a result are not depicted.

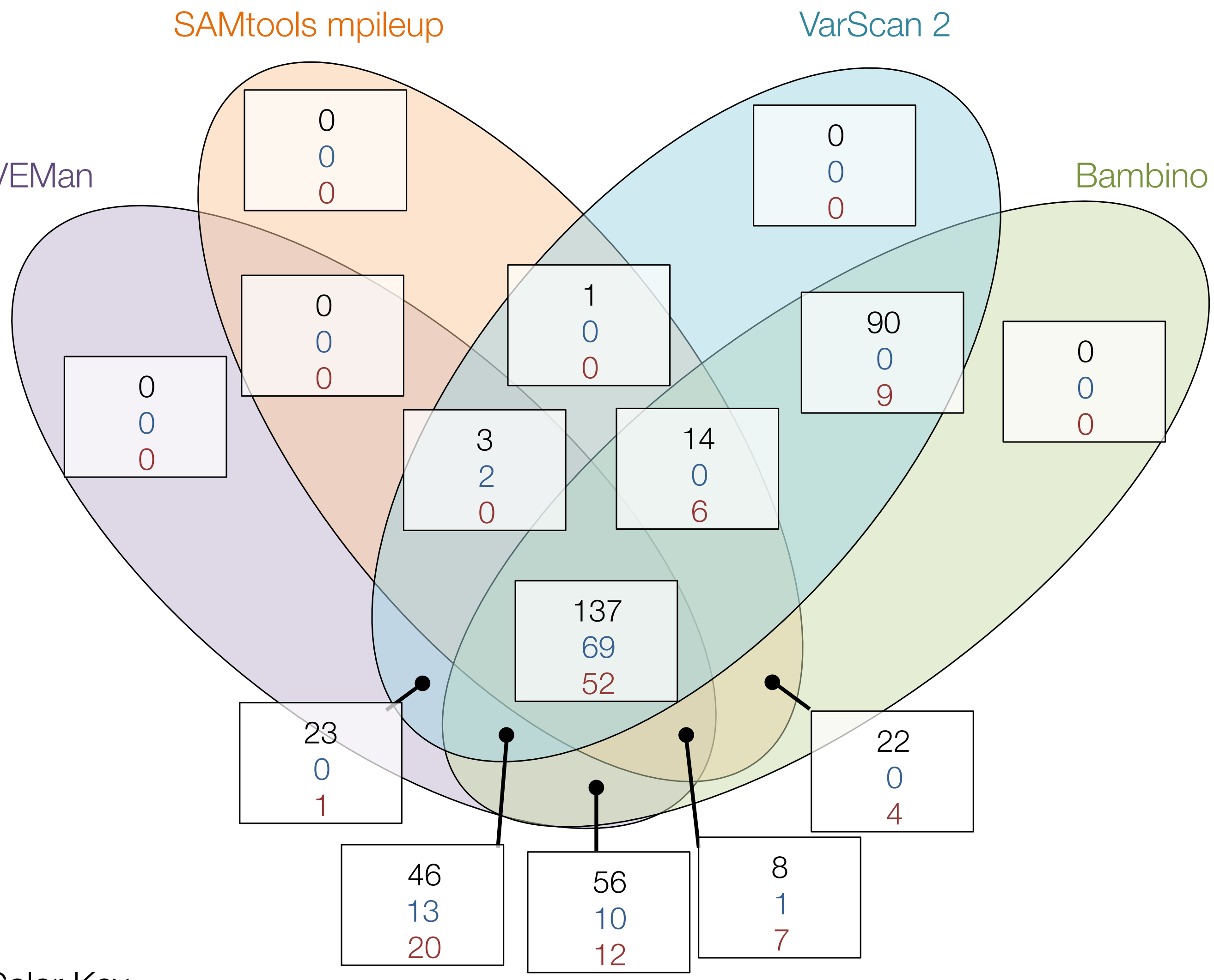

Color Key

Black = Somatic variants sent for Sequenom validation (includes some variants validated in original study)
Blue = Sequenom validated variants which were also validated in the original study
Red = Sequenom validated novel somatic variants (not validated in original study)

Supplementary Table 1

Human hepatocellular carcinoma data

This table shows the overlap of raw variants called from 24 human hepatocellular carcinoma tumour/germline pairs using the algorithms in the Cake pipeline. No filtering was performed on these data. A subset of these data are displayed graphically in Supplementary Figure 3.

Algorithms	Number of overlapping variants	Total number of variants	Percentage overlap	Percentage of validated variants
CaVEMan & VarScan 2	1,943,761	4,438,557	43.80%	94.5%
CaVEMan & mpileup	3,131,603	7,690,925	40.72%	85%
mpileup & VarScan 2	1,845,827	6,859,437	26.90%	86%
Bambino & CaVEMan	2,747,170	30,350,663	9.05%	86.3%
Bambino & mpileup	2,554,243	32,866,536	7.77%	78.6%
Bambino & VarScan 2	1,877,021	29,103,548	6.45%	86.8%
Cake - ≥ any 2 out of 4	3,743,919	33,713,664	11.11%	96.4%
Cake - ≥ any 3 out of 4	2,680,836	33,713,664	7.95%	94.3%
Cake – 4 out of 4	1,664,678	33,713,664	4.94%	77.4%

Supplementary Table 2

General user guidelines for algorithm intersection strategy to use with Cake

	≥ any 2 out of 4 algorithms intersection	≥ any 3 out of 4 algorithms intersection	4 out of 4 algorithms intersection
Targeted gene follow-up (with prior biological hypothesis)	✔		
Large mutation sets for landscape discovery		✔	✔
Number of genes	Dozens	Hundreds/thousands	Whole genome/exome
Smaller sample cohort	✔		
Large sample cohort		✔	✔
Follow-up Validation (Capillary sequencing / Sequenom MassArray)		✔	✔
Follow-up Validation (454 pyrosequencing)	✔		

Supplementary Table 3

Breakdown of novel missense variants called by Cake, human breast cancer data

Somatic Variant	Gene symbol	Consequence	Status in the Stephens, *et al* (2012) study	Disease
1:8418341	*RERE*	Missense	Not seen	leukemia, squamous cell carcinoma
21:47810651	*PCNT*	Missense	Not seen	breast carcinoma
12:122517135	*MLXIP*	Missense	Seen but filtered	
17:55183040	*AKAP1*	Missense	Seen but filtered	prostate cancer
19:51413945	*KLK4*	Missense	Seen but filtered	breast carcinoma
X:153276548	*IRAK1*	Missense	Seen but filtered	non-small cell lung carcinoma

Additionally, we validated 15 more somatic variants, found in non-coding transcripts, that were seen by CaVEMan but filtered out.
